# Systemic therapy in the management of metastatic or advanced salivary gland cancers

**DOI:** 10.1186/1758-3284-4-19

**Published:** 2012-05-04

**Authors:** Aymen Lagha, Nesrine Chraiet, Mouna Ayadi, Sarra Krimi, Bassem Allani, Hela Rifi, Henda Raies, Amel Mezlini

**Affiliations:** 1 Department of medical oncology, Salah Azaiez Institute, Boulevard du 9 Avril, Bab Saadoun Tunis, 1006, Tunisia

**Keywords:** Review, Salivary glands, Adenocystic, Adenocarcinoma, Mucoepidermoid, Treatment, Chemotherapy, Targeted therapy, Hormonotherapy

## Abstract

Salivary gland cancers are very rare tumors. They are characterized by a histologic heterogeneity and a poor outcome. According to this rarity, few prospective data are available to date. No standard recommendations could be held for the use of systemic therapy in these tumors. Several case reports and small studies have investigated the contribution of different agents of chemotherapy. With the extension of molecular biology approach in oncology several signaling pathways have been discovered in different cancers including salivary gland cancers; thus a number of targeted therapies have been investigated. This paper reviewed exhaustively the studies investigating the role of systemic therapies (chemotherapy, targeted therapy, hormone therapy) in salivary gland cancers.

## Introduction

Salivary gland cancers (SGC) are uncommon, accounting for less than 5% of all cancers of the head and neck [1]. The annual incidence rates in the world vary between slightly less than 2 and greater than 0.05 per 100,000 inhabitants [2]. SGC vary considerably in their histologic patterns and behavior. They are classified according to the 2005 World Health system [3], which lists 24 different histologic subtypes. SGC have been characterized by slow growth, multiple local recurrences, and prolonged clinical course, often with the delayed development of distant metastasis [4,5]. In fact, up to 10% of patients who develop metastatic disease survive for more than 10 years [6], and in some cases patients suffer rapid progression and die due to their cancer, highlighting the need for effective therapies, especially chemotherapy or target therapy because surgery or radiotherapy are reserved to treat localized disease. Due to the rarity of this disease and its protracted course, it is difficult to prospectively observe patients with such tumors, and there are few clinical trials investigating the efficacy of systemic therapy. These trials are also very heterogeneous and collectively have enrolled only a small number of patients.

The aim of this paper is to review the role of different agents of chemotherapy, and more recently, the various molecular targets in the management of advanced or metastatic SGC, according to the three most common histologic subtypes: adenoid cystic carcinoma (ACC), adenocarcinoma (ADC) and mucoepidermoid carcinoma (MEC).

## Method

Pub Med, Medline, Cancerlit, Embase, Ovid online, Wiley online and Cochrane were used to search for publications in english relevant to the review. Articles, abstracts, and review articles were identified and reviewed. The reference lists from these sources were searched for additional trials. The last search was done February 1, 2012.

### Chemotherapy

The natural history of metastatic disease is variable, and some patients remain asymptomatic for protracted periods of time. The goal of treatment of metastatic salivary gland tumors is usually palliation, since there is no clear evidence that survival is prolonged by systemic therapy. Watchful waiting may be the most appropriate strategy for patients with indolent disease and few or no symptoms. Systemic therapy may be reserved for those with symptoms and/or rapid disease progression and for those whom local therapy, such as radiation or metastasectomy, is not appropriate. There are limited clinical trials that define the role of systemic therapy in the palliative management of SGC. These include some phase II trials in adenoid cystic cancer and retrospective reports of institutional experiences, but prospective data for the other histologies are scarce, and limited data suggest there are differences in chemotherapy sensitivity among the histologic subtypes of salivary gland tumors. Currently, there are no phase III trials.

#### Monotherapy

All studies with single agent chemotherapy are summarized in (Table 1).

**Table 1:** Studies of single agents in ACC, MEC and ADC of salivary glands

**Treatment**		**ACC**		**MEC**		**ADC**	
**Author**	**Regimen**	**Nb of patients**	**Nb of objective responses**	**Nb of patients**	**Nb of objective responses**	**Nb of patients**	**Nb of objective responses**
**Licitra et al**^**7**^	**Cisplatin 100 mg/m² every 3w**	**13**	**2**	**5**	**1**	**5**	**0**
**DeHann et al**^**9**^	**Cisplatin 50–120 mg/m² every 4w**	**10**	**0**	**-**	**-**	**-**	**-**
**Suen et al**^**13**^	**Cisplatin**	**14**	**9**	**-**	**-**	**-**	**-**
**Kaplan et al**^**14**^	**Cisplatin 100 mg/m²**	**17**	**10**	**-**	**-**	**-**	**-**
**Jones et al**^**40**^	**Cisplatin 100 mg/m²**	**2**	**0**	**2**	**1**	**3**	**0**
**Schramm et al**^**8**^	**Cisplatin 80-100 mg/m² every 4–6 w**	**10**	**7**	**-**	**-**	**-**	**-**
**Mattox et al**^**10**^	**Mitoxantrone 12 mg/m² every 3w**	**18**	**1**	**-**	**-**	**-**	**-**
**Verweij et al**^**11**^	**Mitoxantrone 14 mg/m² every 3w**	**32**	**4**	**-**	**-**	**-**	**-**
**Vermoken et al**^**12**^	**Epirubicin 30 mg/m² weekly, if no response, 90 mg/m² every 3 w.**	**20**	**2**	**-**	**-**	**-**	**-**
**Suen et al**^**13**^**, Kaplan et al**^**14**^	**Doxorubicin (various schema)**	**7**	**3**	**-**	**-**	**-**	**-**
**Airoldi et al**^**15**^	**Vinorelbine 30 mg/m² weekly**	**13**	**2**	**-**	**-**	**5**	**2**
**Spiers et al**^**16**^**Tannock et al**^**17**^	**Cyclophosphamide 100 mg/m² every 3 w**	**7**	**0**	**0**	**0**	**0**	**0**
**Tannock et al**^**17**^**, Suen et al**^**13**^**, Kaplan et al**^**14**^	**5-Fluorouracil (various schema)**	**38**	**16**	**-**	**-**	**-**	**-**
**Tannock et al**^**17**^**, Suen et al**^**13**^**, Kaplan et al**^**14**^	**Methotrexate (various schema)**	**10**	**0**	**5**	**2**	**1**	**0**
**Gilbert et al**^**18**^	**Paclitaxel 200 mg/m² every 3 w.**	**14**	**0**	**14**	**3**	**17**	**5**
**Raguse et al**^**19**^	**Docetaxel 100 mg/m² every 3 w**	**-**	**-**	**4**	**4**	**-**	**-**
**Van Herpen et al**^**21**^	**Gemcitabine 1250 mg/m² d1 and d8 every 3 w.**	**21**	**0**	**-**	**-**	**-**	**-**

ACC = adenoid cyctic carcinoma, MEC = mucoepidermoid carcinoma, ADC = adenocarcinoma.

##### Cisplatin

is the most commonly studied agent in monotherapy. It has been reported to provide a response rate up to 70% in case series less than 15 cases [7,8] . Such results should be interpreted cautiously as they may overestimate the true response rate due to the small number of cases and publication bias in favor of positive results. In contrast to these favorable results, no objective responses were recorded in a study conducted by DeHaan et al. [9] including 10 patients with advanced ACC treated with single-agent cisplatin. In fact, according to DeHaan, the role of cisplatin in SGC remains questionable. More recently, Licitra et al. [7] reported, in the largest phase II study of 25 patients treated with cisplatin in monotherapy,that the response rate for those patients who received and those who did not receive anterior chemotherapy varied between 16% and 21% respectively. For those with metastatic or locoregional disease, the rate varied between 7% and 18% respectively [7]. Differences in responsiveness of histologic types have been claimed, but no conclusions could be drawn due to the lack of sufficient studies. Also, response duration is still too short, falling between 5 and 9 months [7]. Table 1 summarizes the reported studies with cisplatin in monotherapy.

##### Mitoxantrone

Based on its activity in case reports, mitoxantrone was evaluated in 2 phase II studies in adenoid cystic carcinoma. In the first study, Mattox et al. [10] observed only one complete response and 12 stable diseases lasting for 3 months among 18 patients. According to this study, there was no significant anti-tumor activity of mitoxantrone, but we have to keep in mind that all patients of this group were previously treated with surgery, radiotherapy and chemotherapy for an aggressive disease. In 1996, Verweij et al. [11] demonstrated a modest activity of mitoxantrone in adenoid cystic carcinoma. Their study included 32 patients with ACC. Four of them had a partial response lasting from 3 to 13 months and 22 patients had a stable disease. Mitoxantrone was also well-tolerated, except for leucocytopenia observed in 97% of patients.

##### Epirubicin

In a phase II trial including 20 patients with advanced or recurrent ACC treated with epirubicin, Vermorken et al. [12] reported a low objective response rate (10%) and a rapid improvement in symptoms-related disease in 5 patients (29.4%). However, median time to progression was too short (16 weeks) and the median survival was about 60 weeks. Some authors have reported as well that local or regional control with anthracyclines appears to be superior to metastatic control [12-14].

##### Vinorelbin

As single agent, vinorelbin is reported to have moderate activity in ACC and adenocarcinoma [15]. It is well tolerated and its activity is similar to that reported for cisplatin, 5-Fluorouracil (5FU), and anthracyclines. In a study conducted by Airoldi et al. [14], the overall response rate for patients treated with vinorelbin alone was 20% (4 of 20 patients), the median duration of partial response was 6 months (range, 3-9 months), the median stable disease duration was 3.5 months (range, 2–10 months), the median time to disease progression was 5 months, and the median overall survival duration was 8.5 months (range, 2.5-10 months). The poor results reported most likely are due to the high percentage of adenoid cystic carcinoma, as well as to the fact that all patients had been heavily treated previously and all tumor recurrences were bulky and progressive.

##### Cyclophosphamide

It was evaluated in ACC in two small series [16,17] without any objective responses reported, except for 2 instances of 6 and 39 months of stationary disease among 9 patients. But, its significance is questionable in a tumor that is well known for its frequently indolent course and slow growth, and it may have played no part in the stabilization of such disease.

##### 5-Fluorouracil

5FU appeared to be active in ACC as a single agent. Kaplan et al. [14] and Suen et al. [13] reported in 2 different studies a response rate of 46% (6 objective responses among 13 patients). Tannock et al. [17] also reported in 1980, 4 objective responses among 12 patients. Duration of response ranged from 5 to 24 months.

##### Methotrexate

Based on its activity in epidermoid carcinoma of the head and neck, Methotrexate (MTX) was evaluated in SGC, with 2 partial responses in muco-epidermoid carcinoma among 5 patients [13,14]. However, MTX did not show any anti-tumor activity in the other histologic subtypes especially ACC and adenocarcinoma [13,14,17].

##### Paclitaxel

In phase II evaluation of single-agent paclitaxel in SGC, Gilbert et al. [18] reported a modest response rate in patients with muco-epidermoid and adenocarcinoma histologic subtypes, noting 3 objective responses among 14 patients and 5 objective responses among 17 patients respectively. No responses were seen in the adenoid cystic carcinoma group. The poor response rate in ACC was consistent with prior reports in this chemoresistant histologic subtype. Gilbert et al. concluded that there was a trend towards a difference in sensitivity to paclitaxel among the histologic subtypes: muco-epidermoid and adenocarcinoma versus adenoid cystic carcinoma.

##### Docetaxel

Based on its impressive anti-tumor activity in patients with head and neck cancer, especially in squamous cell carcinoma, Ragusa et al. [19] evaluated its activity in 4 patients with high grade muco-epidermoid carcinoma of the major salivary glands. The treatment was well tolerated, and there was complete response in two and partial response in the other two patients. Docetaxel seems to be a logical alternative in muco-epidermoid SGC, but at this time there only a few reports [19,20] and the true activity of docetaxel may be overestimated due to publications bias in favor of positive results.

##### Gemcitabin

Van Herpen et al. [21] evaluated the antitumor activity of gemcitabin in ACC in a phase II study including 21 patients. Therapy was well tolerated but no objective response was reported. Thus, gemcitabin is ineffective in such tumors.

#### Polychemotherapy

**Table 2:** Studies of combination chemotherapy in ACC, MEP and ADC of salivary glands

**Treatment**		**Adenoid cystic carcinoma**		**Mucoepidermoid carcinoma**		**Adenocarcinoma**	
**Author**	**Regimen**	**Nb of patients**	**Nb of objective responses**	**Nb of patients**	**Nb of objective responses**	**Nb of patients**	**Nb of objective responses**
**Kaplan et al**^**14**^	**C :200 mg/m² d3-d6 A :40 mg/m² d1 P:50-100 mg/m² d1 every 4 w.**	**-**	**-**	**2**	**1**	**4**	**4**
**Alberts et al**^**22**^	**C :200 mg/m² d3-d6 A :40 mg/m² d1 P :50 mg/m² d1 every 4 w.**	**-**	**-**	**-**	**-**	**3**	**3**
**Dreyfuss et al**^**23**^	**C : 500 mg/m² d1 A :50 mg/m² d1 P : 50 mg/m² d1 every 4w.**	**9**	**3**	**-**	**-**	**4**	**3**
**Belani et al**^**24**^	**C : 400 mg/m² d1 A: 40 mg/m² d1 P : 60 mg/m² d1 every 3–5 w.**	**4**	**1**	**3**	**3**	**1**	**1**
**Creagan et al**^**25**^	**CAP (various schema)**	**11**	**2**	**7**	**2**	**14**	**7**
**Licitra et al**^**26**^	**C: 500 mg/m² d1 A: 50 mg/m² d1 P: 50 mg/m² d1 every 3w.**	**12**	**3**	**1**	**0**	**2**	**0**
**Eisenberg et al**^**27**^	**CAP(not mentioned)**	**0**	**0**	**3**	**2**	**1**	**1**
**Venook et al**^**28**^	**P :50 mg/m² A :30 mg/m² F :500 mg/m² all days1,8 every 4 weeks**	**9**	**3**	**4**	**2**	**3**	**2**
**Dimery et al**^**29**^	**C :500 mg/m² d1 A :50 mg/m² d1 P:40 mg/m² d2d3 F:500 mg/m² d1d2 every 3–4 w.**	**7**	**3**	**1**	**1**	**9**	**4**
**Airoldi et al**^**30**^	**P :60 mg/m² d1 E :50 mg/m² d1 F :600 mg/m² d1 every 3 w.**	**4**	**2**	**-**	**-**	**2**	**1**
**Ross et al**^**31**^	**P :60 mg/m² d1 E :50 mg/m² d1 F :200 mg/m² d1 every 3 w.**	**8**	**1**	**-**	**-**	**-**	**-**
**Tsukuda et al**^**32**^	**C :400 mg/m² d1 Pr :40 mg/m² d1 P :60 mg/m² d1 every 4 weeks**	**6**	**2**	**-**	**-**	**8**	**3**
**Kaplan et al**^**14**^	**C:200 mg/m² d3-d6 A:40-60 mg/m² d1 every 3 w.**	**5**	**2**	**3**	**0**	**3**	**1**
**Posner et al**^**33**^	**C :450 mg/m² d1 A :45mh/m² d1 every 3w**	**5**	**2**	**3**	**0**	**-**	**-**
**DeHaan et al**^**9**^	**P:20 mg/m² d1-d5 A:50 mg/m² d1 B :30 mg d1-d5 every 3w.**	**9**	**3**	**-**	**-**	**-**	**-**
**Triozzi et al**^**34**^	**C :1000 mg/m² d1 Vn :1 mg d1,d4 F :750 mg/m² d1-d4 every 4w.**	**8**	**2**	**-**	**-**	**-**	**-**
**Hill et al**^**35**^	**P :100 mg/m² d1 F :1 g/m² d1-d4 every 4w.**	**11**	**0**	**-**	**-**	**-**	**-**
**Airoldi et al**^**15**^	**P:80 mg/m² d1 V:25 mg/m² d1d8 every 3 w.**	**9**	**4**	**1**	**0**	**4**	**3**
**Airoldi et al**^**36**^	**Cb:AUC5 d1 T :175 mg/m² d1 every 3w.**	**10**	**2**	**1**	**0**	**1**	**0**
**Ruzich et al**^**37**^	**Cb :AUC6 d1 T :200 mg/m² d1 every 3w.**	**-**	**-**	**-**	**-**	**1**	**1**
**Gedlicka et al**^**38**^	**P :30 mg/m² d1-d3 M:12 mg/m² d1 every 3w.**	**11**	**1**	**-**	**-**	**-**	**-**
**Laurie et al**^**39**^	**G :1000 mg/m² d1d8 P : 70 mg/m² d2 or Cb : AUC5 d1 every 3w.**	**10**	**2**	**4**	**1**	**8**	**3**
**Posner et al**^**33**^	**P:20 mg/m² d1-d5 B:10 mg/m² d3-d7 Mtx : 200 mg/m² d14d21 every 3w.**	**-**	**-**	**3**	**2**	**-**	**-**
**Kaplan et al**^**14**^	**P :80 mg/m² d1 B :10 mg/m² d1 Mtx : 40-60 mg/m² d1**	**2**	**0**	**1**	**0**	**-**	**-**
**Jones et al**^**40**^	**E :75 mg/m² d1 F :100 mg/m² d1 every 3w**	**3**	**0**	**1**	**0**	**3**	**0**
**Pedani et al**^**41**^	**P:60 mg/m² d1 I:60 mg/m² d1d8 every 3 w**	**10**	**1**	**1**	**0**	**1**	**0**

P = cisplatin. F = 5-fluorouracil. A = doxorubicin. B = bleomycin. C = cyclophosphamide. Vn = vincristine. E = epirubicin. Cb = carboplatin. AUC = area under the curve. Pr = pirabucin. V = vinorelbine. T = paclitaxel. M = Mitoxantrone. G = gemcitabine. I = irinotecan. Mtx = methotrexate.

Based on the activity of some chemotherapeutic agents previously cited, some combination chemotherapy regimens, which include 2 or more drugs, have been evaluated in patients with advanced or recurrent SGC (all studies with polychemotherapy are summarized in Table 2).

##### CAP (Cyclophosphamide/Doxorubicin/Cisplatin)

The most commonly studied regimen, CAP (cyclophosphomide, doxorubicin, cisplatin) has been reported as an active regimen in SGC [14,22-27]. In fact, Alberts et al. [22] observed responses that included 2 complete remissions in all five of their patients. Kaplan et al. [14] reported 5 objective responses including 1 complete response among 6 patients. As well, Dreyfuss et al. [23] reported 3 complete and 3 partial remissions among 13 patients. In a study conducted by Creagan et al. [25] including 34 patients treated with 3 different CAP regimens, 2 complete and 11 partial responses were reported. Another small but enlightening trial was composed of four patients of whom three achieved, with CAP**,** complete responses, lasting up to 12 months [27]. Also, Belani et al. [24] treated 8 patients with CAP, resulting in 3 complete and 2 partial responses. Licitra et al. [26], in a phase II trial of 22 patients treated with CAP, reported that only six patients achieved partial responses with an overall response rate of 27%. When all these studies are combined, the overall response rate to CAP is 46%. Combined, 43 of 92 patients with advanced, recurrent or metastatic SGC have responded to CAP; 15 of these patients achieved complete remissions, and duration of response averages were between 3 and 72 months. Based on the incidence of objective response, CAP appears to be more active than single-agent therapy; however these results should be interpreted cautiously since they represent patients from various trials using different doses and schedules, involving as well a small number of patients.

##### PAF (Cisplatin/Doxorubicin/5FU)

The 3 most effective chemotherapeutic agents in SGC have been evaluated by Venook et al. [28] in a combination regimen that included cisplatin, doxorubicin and 5-fluorouracil. Seventeen patients with advanced or recurrent SGC were included in this pilot study. Only 2 patients achieved a complete response (12%), and 4 achieved a partial response (23%); for an overall response rate of 35%. The response duration was 6 to 15 months. There was no difference in response between those patients treated for metastatic/recurrent disease, nor among the different histologic subgroups. In conclusion, despite the lack of survival advantage in this study, PAF chemotherapy can be administered on an outpatient basis and clearly has a role in the palliation of metastatic and/or recurrent SGC, particularly in adenocarcinoma.

##### CAPF (Cyclophosphamide/Doxorubicin/Cisplatin/5FU)

Based on the results of the aforementioned drugs, cisplatin, doxorubicin, cyclophosphamide and 5-fluorouracil have been reported as having the best anti-tumor activity in SGC. In a study involving 17 patients and conducted by Dimery et al. [29], only one patient achieved complete response (6%) and seven other patients (44%) had a partial response, for a total objective response rate of 50%. The median duration of all objective responses was 7 months (range between 1 and 16 months). Additionally, this regimen entailed an increased risk of hematologic and non hematologic toxicity (2 drug-related deaths). Moreover, the objective response rate in this trial is consistent with other reports of combinations containing doxorubicin and cisplatin, therefore the addition of cyclophosphamide and 5 FU does not indicate an improved benefit. In conclusion, despite the very aggressive four-drug combination, which used the perceived most active agents at maximal dosage, as evidenced by its toxicity, the response rate was not substantially increased in comparison with other studies.

##### PEF (Cisplatin/Epirubicin/5FU)

In view of the relative efficiency of the CAP regimen in SGC and the lower systemic and cardiac toxicity of epirubicin compared to the parent compound doxorubicin, a new regimen containing cisplatin, epirubicin and 5-fluorouracil has been evaluated in 2 different studies. In the first one, Airoldi et al. [30] reported one complete response (11%) and 3 partial responses (33%) among 9 patients with a median response duration of 7.5 months. In the second study conducted by Ross et al. [31], which included 8 patients, only one patient achieved a partial response. This regimen was generally well tolerated and there were no treatment-related deaths in both studies. The objective response rate was 29%, and patients with local recurrence showed a better response than patients with local and distant recurrences [30]. In conclusion, the activity of PEF in SGC does not seem to be superior to previously reported phase II trials of CAP.

##### CPPr (Cyclophosphamide/Cisplatin/Pirabucin)

According to the results of CAP, the combination of an anthracycline with these two alkylating agents seems to be the most effective. However, anthracyclines entail severe side effects specially cardiotoxicity. Pirarubicin is an analogue of the anthracycline antineoplastic doxorubicin known as less cardiotoxic than doxorubicin and exhibiting activity against some doxorubicin-resistant cell lines. Tsukuda et al. [32] reported a response rate of 36% (5/14) in a study including 14 patients with adeno or adenocystic carcinoma of the salivary glands treated with a combination chemotherapy regimen of cyclophosphamide, pirarubicin and cisplatin. The median duration of response was 37 months in the one complete response case and 16 months (range, 6 to 20) in the 4 partial response cases. The severity of the side-effects was less with this scheme than a CAP regimen. This regimen may be an interesting alternative for CAP but needs more studies for confirmation.

##### CA (Cyclophosphamide/Doxorubicin)

Posner et al. [33] reported 5 objective responses (38%) among 13 patients treated with CA (cyclophosphomide-Doxorubicin) regimen. Kaplan et al. [14] showed a response rate of 33% (5 partial responses among 15 patients). The overall objective response was 35% in both reports. CA regimen was well tolerated and effective in patients with all histologic subtypes except muco-epidermoid carcinoma.

##### PAB (Cisplatin/Doxorubicin/Bleomycin)

A chemotherapeutic regimen based on cisplatin, doxorubicin and bleomycin was tested by DeHann et al. [9] in one prospective study including 9 patients with ACC. The observed toxicity -essentially myelotoxicity- was acceptable. Three objective responses (33%) were observed: one complete remission and 2 partial remissions. The response duration ranged between 6 and 21 months. In conclusion, this regimen is to some extent comparable with the CAP regimen.

##### CFVn (Cyclophosphamide/5FU/Vincristin)

A non-cisplatin based regimen (cyclophosphamide, vincristin and fluorouracil) was tested in a study including 8 patients with ACC of the salivary glands [34]. This regimen was effective: objective response rate was 25% (2 partial responses). However, no conclusions can be drawn due to the small number of patients included in this study.

##### PF (Cisplatin/5FU)

Hill et al. [35] evaluated the association Cisplatin/5-fluorouracil in 11 patients with advanced ACC. No objective responses were observed, but the symptomatic response rate was 64% and toxicity was manageable. Therefore, PF could be a useful palliative regimen in cases of symptomatic ACC.

##### PV (Cisplatin/Vinorelbine)

In a phase II trial including 16 patients with recurrent SGC, Airoldi et al. [15] evaluated the cisplatin-vinorelbine combination. Three complete and 4 partial responses were noted and the overall response rate was 44%. The median duration of the CR was 15 months (range, 6–27 months); for PR, the median duration was 7.5 months (range, 3–11 months). In comparison to CAP, PV appears to be a less toxic regimen, and the results are nearly comparable.

##### PT (Carboplatin/Paclitaxel)

Airoldi et al. [36] reported 2 partial responses (14%) lasting 5 and 12 months in a study including 14 patients with recurrent SGC treated with carboplatin/paclitaxel combination. The treatment was well tolerated. Ruzich et al. [37] also observed one complete response in metastatic SGC treated with the same regimen. These results suggest that PT regimen has moderate activity in SGC, but it is reasonable to consider further evaluation of this combination in future phase II trials.

##### PM (Cisplatin/Mitoxantrone)

Mitoxantrone is an anthraquinone antineoplastic agent with structural and functional similarities to anthracyclines. Because it is less cardiotoxic than anthracyclines and analogous to the CAP regimen, Gedlicka et al. [38] evaluated the efficacy of Cisplatin/Mitoxantrone combined in a phase II trial involving 14 patients with recurrent or metastatic SGC. The response to treatment was PR in two patients (response duration 27 and 14 months) yielding an overall response rate of 14.3%. With regard to tolerance, myelosuppression was commonly observed (grade 3 or 4 in 60%). In conclusion, no additional benefit has been yielded with the cisplatin/mitoxantrone combination compared to CAP but it seems more myelotoxic.

##### GP (Gemcitabin/Cisplatin)

Laurie et al. [39] evaluated the combination of gemcitabin with cisplatin (or carboplatin) in a phase II study including 33 patients with advanced SGC. Toxicity was within that expected for this combination, and 8 objective responses were observed (1 complete and 7 partial) for a response rate of 24%. The duration of response ranged from 1.3 to 11. 3 months, with a median of 6.7 months. As well, responses were observed in all common histologic subtypes. This regimen may have promising activity in patients with adenocarcinoma histology. Given the absence of responses in patients treated with carboplatin in this trial due to impairment of renal function or hearing deficit, Laurie et al. do not support the routine substitution of carboplatin for cisplatin in the treatment of advanced SGC. Moreover, GP regimen does not offer an advantage over other cisplatin-based regimens particularly CAP or single-agent cisplatin. In conclusion, this combination demonstrated some modest activity in advanced SGC.

##### EF (Epirubicin/5FU)

A non-cisplatin based regimen (epirubicin and fluorouracil) was tested in a phase II trial involving 7 patients with advanced SGC [40]. There was no response and the median survival was 8 months.

##### PBMtx (Cisplatin/Bleomycin/Methotrexate)

Posner et al. [33] reported 2 objective responses among 3 patients with MEC, but the duration of responses was too short (2 and 4 months). However, Kaplan et al. [14] did not report objective response in 3 patients with advanced SGC. Therefore, no conclusion can be drawn due to the small number of patients and heterogeneity of the results.

##### PI (Cisplatin/Irinotecan)

Fourteen patients with advanced SGC were treated with the combination Cisplatin/Irinotecan in a study conducted by Peldani et al. [41]. Only one partial response (7%) lasting 4 months was reported. This regimen was not well-tolerated. Grade 3–4 neutropenia and diarrhea was noted in 9 (64%) and 4 (28%) patients, respectively. Thus, PI regimen was less effective and more toxic than other combination such as PV or PT.

### Targeted therapy

**Table 3:** Studies of molecular targeted therapies in ACC, MEC and ADC of salivary glands

**Treatment**		**Adenoid cystic carcinoma**		**Mucoepidermoid carcinoma**		**Adenocarcinoma**	
**Author**	**Regimen**	**Nb of patients**	**Nb of objective responses**	**Nb of patients**	**Nb of objective responses**	**Nb of patients**	**Nb of objective responses**
**Guigay et al**^**49**^	**Imatinib 400 mg twice daily**	**17**	**2**	**-**	**-**	**-**	**-**
**Slevin et al**^**50**^	**Imatinib 800 mg daily for 2 months, if stable disease: P 80 mg/m² every 3w + Imatinib 400 mg daily**	**18**	**0**	**-**	**-**	**-**	**-**
**Ochel et al**^**51**^	**Imatinib 400 mg daily**	**4**	**0**	**-**	**-**	**-**	**-**
**Lin et al**^**52**^	**Imatinib 400 mg twice daily**	**5**	**0**	**-**	**-**	**-**	**-**
**Hotte et al**^**53**^	**Imatinib 40 mg twice daily**	**16**	**0**	**-**	**-**	**-**	**-**
**Pfeffer et al**^**54**^	**Imatinib 400 mg daily, with possible escalation to 800 mg**	**10**	**0**	**-**	**-**	**-**	**-**
**Alcedo et al**^**55**^	**Imatinib 400-600 mg daily**	**2**	**2**	**-**	**-**	**-**	**-**
**Faivre et al**^**56**^	**Imatinib 800 mg daily**	**8**	**0**	**-**	**-**	**-**	**-**
**Glisson et al**^**58**^	**Gefitinib 250 mg daily**	**19**	**0**	**2**	**0**	**3**	**0**
**Agulnik et al**^**59**^	**Lapatinib 1500 mg daily**	**19**	**0**	**2**	**0**	**7**	**0**
**Locati et al**^**60**^	**Cetuximab 400 mg/m² loading dose, then 250 mg/m² weekly.**	**23**	**0**	**2**	**0**	**1**	**0**
**Haddad et al**^**77**^	**Trastuzumab: 4 mg/kg loading dose, then 2 mg/kg weekly.**	**2**	**0**	**3**	**1**	**7**	**0**
**Argiris et al**^**83**^	**Bortezomib 1.3 mg/m² days 1,4,8,11 every 3 weeks. At progression: doxorubicin 20 mg/m² days 1,8.**	**25**	**0**	**-**	**-**	**-**	**-**

The understanding of the underlying molecular changes in malignant SGC has led to the identification of several potential therapeutic targets. There are few phase II trials evaluating these agents, and the data are too preliminary to recommend their routine use (all studies with target therapies are summarized in (Table 3).

#### Tyrosine kinase inhibitors

##### Imatinib

The c-kit tyrosine kinase (c-Kit TK) receptor is expressed in up to 100% of ACC, and it is associated with the histologically solid subtype [42-47]. It was present in up to 60% of basal cell adenocarcinomas and 50% of basaloid squamous carcinomas [42]. However, the contribution of the c-kit pathway to cell proliferation and carcinogenesis in ACC is unclear, especially because the presence of a specific c-kit mutation has yet to be detected within the small number of ACC studied [42]. Moreover, Oliveira et al. [48] have demonstrated that Kit was expressed but not phosphorylated in a series of four ACC examined. The authors concluded that Kit expression is unlikely to have a direct oncogenic function in ACC, in contrast to its role in gastrointestinal stromal tumors. Therefore, it has been established that c-kit protein overexpression, rather than mutation, is involved in the pathogenesis of ACC. To date, no gene mutation has been clearly identified as the mechanism of c-kit activation in this neoplasm [42-44]. Seven studies have assessed imatinib in over 80 advanced ACC (7 studies have used imatinib alone, and one study has evaluated the association imatinib/cisplatin), with only 4 partial responses, for an objective response rate of 5% [49-56]. The duration of these response was short (range, 9–15 months) [49]. However, stable disease was reported more commonly 29% (21 among 72 patients). In light of this poor result and the lack of c-kit exons mutation to date, the anti-tumor activity of imatinib in ACC remains questionable.

##### Gefitinib

It is well established that over-expression or alteration in epidermal growth factor receptor (EGFR) is involved in the pathogenesis of many tumors [43]. It was reported also, that EGFR overexpression in ACC has varied from none to 85% [1,43,57]. A possible interaction between EGFR and SGC was suggested. Glisson et al. [58] evaluated the efficacy of an orally active EGFR tyrosine kinase inhibitor (gefitinib) in 28 patients with advanced SGC. The treatment was well-tolerated, but no objective responses were documented. However, stable disease was observed in 14 patients (67%) with a median duration of 3 months (range, 1–4.5 months). Given the indolent nature of SGC, stable disease responses become an unreliable end-point for SGC especially for short durations. Thus, interpretation of the value of stable disease requires further investigation.

##### Lapatinib

Agulnik et al. [59] tested lapatinib, a dual inhibitor of the tyrosine kinase domains of EGFR and erbB2, in a phase II study including 40 patients with progressive metastatic or recurrent EGFR/Her2-overexpressing SGC. The treatment was well tolerated. No objective responses were observed, but 13 patients (33%) had stable disease for at least 6 months. In this study, disease progression was required prior to enrollment, thus disease stabilization is more likely due to lapatinib than the relatively indolent natural course of SGC.

#### Monoclonal antibodies

##### Cetuximab

Another anti-EGFR monocolonal antibody (Cetuximab) was evaluated in a phase II study involving 30 patients with recurrent or metastatic SGC [60]. Only 16 patients with documented progressive disease were included in this study. Skin toxicity was the main adverse event. No objective responses were observed, but stable disease was recorded in 24 (80%) patients, 15 (50%) of whom lasted more than six months. Also, Locati et al. [60] reported that neither the EGFR over-expression nor the EGFR copy number nor skin rash were correlated with the treatment outcome. In view of this relative clinical benefit, correlation between EGFR expression and outcome is pending.

##### Trastuzumab

Several studies have evaluated the overexpression of c-ErbB2 (HER2/neu) in SGC, by either immunostaining or fluorescence in situ hybridization. According to the different histologic subtypes, there is a large disparity in the proportion of SGC that overexpresses HER2. In fact, this rate ranged from 0% to 58% in adenoid cystic [61-70], from 14% to 39% in adenocarcinoma [64,67-69], from 0% to 38% in muco-epidermoid [61,64,68,69,71-74], and from 24% to 100% in terminal duct adenocarcinoma [61,64,75,76]. In view of these studies, expression of HER2 is unusual in cancers of intercalated duct origin (adenoid cystic, adenocarcinoma, acinic cell, polymorphous low-grade adenocarcinoma, and myoepithelial), when compared with those derived from the secretory duct (salivary duct, mucoepidermoid, and squamous). In a phase II trial of trastuzumab -an anti HER2 monocolnal antibody- that included 14 patients with advanced SGC overexpressing Her2/neu, Haddad et al. [77] identified only one partial response lasting longer than 2 years in MEP. Also, Nabili et al. [76] reported a complete response lasting for 3 years in one patient among three with progressive salivary duct carcinoma treated with trastuzumab. There are some case reports as well of lasting responses to trastuzumab in salivary duct carcinoma [78-80]. In conclusion, the clinical utility of trastuzumab therapy in SGC of intercalated duct origin is very limited; however, this agent has showed reasonable activity in excretory duct subtypes.

#### Proteasome inhibitor

##### Bortezomib

The nuclear factor kappa-B (NF-kappa B), a key target of bortezomib, is expressed in ACC, and is related to angiogenesis and poor patient outcome [81,82]. In theory, the inhibition of NF-kappa-B activity can suppress the growth of SGC. Argiris et al. [83] evaluated the activity of bortezomib in a phase II trial including 25 patients with advanced ACC. The treatment was well tolerated. There were no complete or partial responses from bortezomib in monotherapy, but fifteen patients (71%) showed disease stabilization for a median duration of 4.2 months (range: 0–20.1 months). Also, among 10 patients who received bortezomib plus doxorubicin in this study, 1 had a partial response, and 6 had stable disease with a median duration of 5.22 months (range: 0–10 months). In conclusion, the role of bortezomib in SGC needs further investigation.

### Hormone therapy

Usually SGC did not express hormone receptors [84-87]. In contrast, it has been reported that some SGC possess hormonal receptors, such as estrogen [88] or progesterone [89-92] or even androgen receptors in salivary duct carcinoma [92]. In light of these reports, hormonal treatment has been used occasionally, and isolated case reports document objective responses in patients with ACC treated with tamoxifen [93,94] and in patients with salivary duct cancer and adenocarcinoma treated with antiandrogen therapy [95,96]. To date, no phase II studies have been performed, so it is difficult to define the role of hormone therapy in SGC.

## Discussion

In monotherapy, most of these chemotherapeutic agents have modest activity in the treatment of advanced SGC with response rates between 10 and 70%, and this response is of short duration. However, Cisplatin, Doxorubicin and 5-Fluorouracil appeared to be the most active single agents.

The various studies published up to date are difficult to compare, and identifying the most chemotherapeutic regimen is not easy due to the low incidence of this disease and its disparate histologies. Some authors suggest that chemotherapy for SGC may need to be cell-type specific (eg doxorubicin-containing regimens in ACC and adenocarcinoma [28], Paclitaxel in adenocarcinoma and MEP[18]).

Compared to monotherapy, combination chemotherapy regimens generally result in higher response rates, but they are not clearly superior in survival outcomes despite additional toxicity. An initial platinum and doxorubicin combination is preferred whenever the patient is very symptomatic to maximize the likelihood of a response. Otherwise, a single agent therapy is sufficient.

To date, none of the aforementioned targeted therapies have shown any real anti-tumor activity in SGC. The best obtained responses were disease stabilization for a short period of time (a few months). On the other hand, progression of disease was not required as an inclusion in several studies, and this might limit potential activity of target therapy in terms of response rate.

## Ongoing clinical trials

Currently, 210 trials involving SGC are ongoing [97]. The following chemotherapeutic agents are under study: bleomycin, docetaxel, doxorubicin, capecitabin, carboplatin, cisplatin, epirubicin, gemcitabin, irinotecan, methotrexate, oxaliplatin, paclitaxel, pemetrexed disodium and thalidomide. Some targeted therapies are also under study: Allovectin-7, antineoplaston AS2-1, bevacizumab, bortezomib, cediranib maleate, celecoxib, CNGRC peptide-TNF alpha conjugate, cetuximab, dasatinib, DetoxPC, eribulin mesylate, erlotinib, everolimus, fenretinid, filgrastim, flavopiridol, gefitinib, imatinib mesylate, indinavir sulfate, interleukin-12, ispinesib, ixabepilone, lapatinib, lonafarnib, monoclonal antibody RAV12, panitumumab, Perifosin, romidepsin, ritonavir, semaxanib, sorafenib, sunitinib, talactoferrin, temoporfin, trastuzumab, vandetanib, vorinostat.

The aforementioned drugs are evaluated either alone or in combination or with radiotherapy.

## Conclusion

SGC are characterized by frequent local recurrences and distant metastases consistent with a prolonged survival. The role of systemic therapies in the management of such advanced, recurrent and metastatic tumors still needs to be defined: in fact the most anticancer drugs are active against rapidly proliferating cells, thus the slow growth of SGC could explain the poor results. Also, in the absence of good responses, the need to treat for prolonged periods limits any benefit to be obtained from drugs which demonstrate cumulative toxicity.

Response rates to chemotherapy are too low and usually for a short duration (only a few months), but symptomatic responses seem to be greater. The most effective chemotherapeutic agents seem to be platinum, 5-Fluorouracil and anthracyclines. Therefore, these agents used either in monotherapy or in combination are reasonable first-line options. On the other hand, targeted therapy results have been disappointing, especially the objective responses reported in several studies. However, it has been suggested that the duration of stable disease is probably more important than the objective response rate to conclude that a given target therapy has or not a real anti-tumor activity (such as Trastuzumab, Cetuximab and Lapatinib). Concerning hormonal therapy, the actual data are too limited to draw any conclusions.

To date and in view of the modest activity of all current agents and the natural indolent behavior of SGC, there is no evidence that systemic therapies improve survival; therefore, they should be reserved for relief of disease-related symptoms or for cases of rapid progression. Also, the choice of treatment should be dictated by histologic subtypes, patient characteristics and comorbidities, toxicity and cost of drugs.

At present, further clinical trials with new drugs, new targeted therapies and new combinations to determine better systemic treatment are required.

## Authors’ contributions

All authors have contributed and met the requirements of authorship for this article by assisting in data interpretation, draft editing and revising for important intellectual content, and final approval of this version to be published.

## Competing interests

Authors declare no potential conflicts of interest.
